# A Case of Apparent Life-Threatening Event: Comorbid Gastric Volvulus Associated Gastroesophageal Reflux Disease and Epilepsy in a 4-Month-Old Boy

**DOI:** 10.1155/2016/5717246

**Published:** 2016-05-12

**Authors:** Yoshihiko Takano, Masaki Horiike, Ako Tatsumi, Haruko Sakamoto, Hisanori Fujino, Shin-ichi Sumimoto

**Affiliations:** ^1^Department of Pediatrics, Osaka Red Cross Hospital, 5-30 Fudegasaki-cho, Tennoji-ku, Osaka 543-8555, Japan; ^2^Department of Pediatric Surgery, Osaka Red Cross Hospital, 5-30 Fudegasaki-cho, Tennoji-ku, Osaka 543-8555, Japan

## Abstract

Most isolated episodes of apparent life-threatening events (ALTEs) do not lead to the diagnosis of serious conditions, and their prognoses are generally benign. However, recurrent ALTEs are often associated with a risk of future serious adverse events and should be evaluated for appropriate management. Here we present ALTE case in which gastric volvulus associated gastroesophageal reflux disease was detected as an etiology initially, followed by the detection of epilepsy as another etiology. Clinicians should consider possibility of two or more etiologies in a single recurrent ALTE case.

## 1. Introduction

Apparent life-threatening events (ALTEs) are defined as episodes characterized by a combination of apnea, color change, altered muscle tone, choking, and gagging, which are frightening to the observer [1]. The most common ALTE etiologies are gastroesophageal reflux disease (GERD), epilepsy, and respiratory tract infection, accounting for approximately 50% of cases. However, no definite diagnosis is made in approximately 50% of ALTE cases [2]; therefore, it is rare to have two or more etiologies for a single ALTE case. Here we report ALTE case in which multiple etiologies were diagnosed.

## 2. Case Report

**Figure 1:**
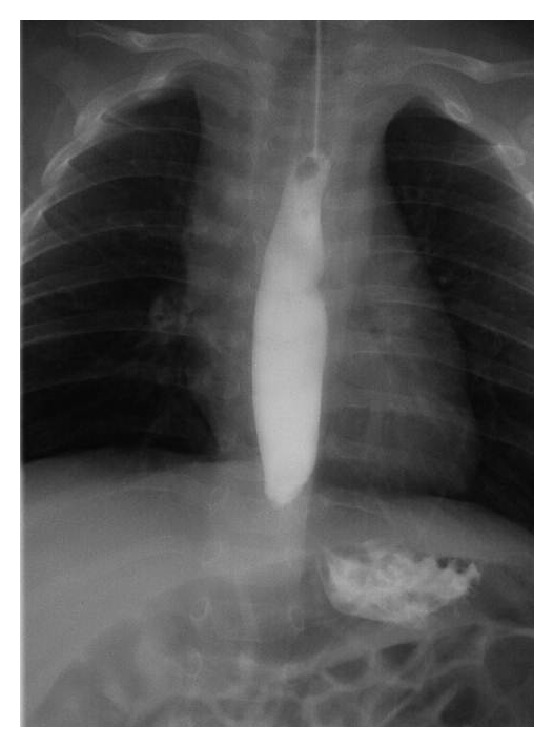
An upper gastrointestinal contrast study revealing an esophageal motility dysfunction. Contrast agent does not flow into the stomach and is seen to pool in the middle to lower esophagus.

**Figure 2:**
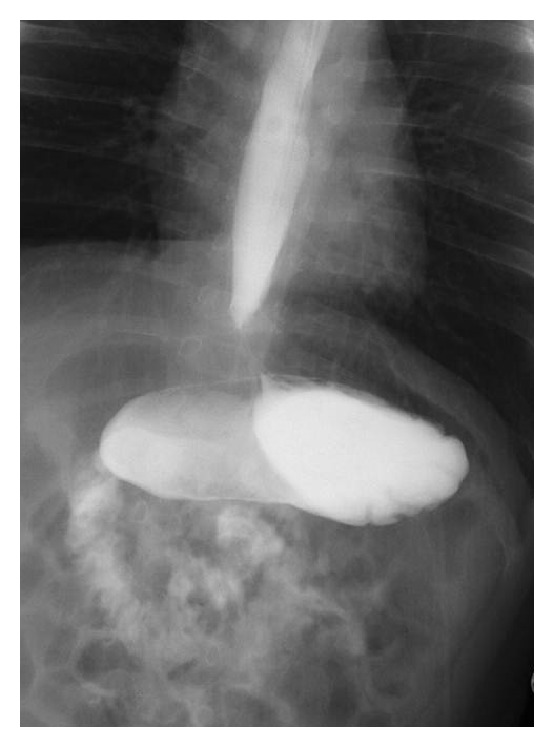
Organoaxial gastric volvulus. The stomach is oriented with the organoaxial (longitudinal) axis which extends from the gastroesophageal junction to the pylorus, thus giving an “upside-down” appearance.

A 4-month-old Japanese boy with a normal perinatal history was brought to our hospital with complaints of cyanosis and hypotonia. He was healthy on physical examination. He had no exposure to passive smoking and there was no index of suspicion for abuse, but his cousin had a history of epilepsy. He was admitted for a thorough evaluation. During the 3-day admission period, he showed no symptoms or attacks. Several test results, including complete blood cell count, biochemistry assessment, venous blood gas, chest X-ray, electrocardiogram, echocardiography, brain CT-scan, short-term electroencephalogram (EEG) for 30 min, lactate/pyruvate levels, and a pertussis test, revealed no abnormalities. Three days after getting discharged from the hospital, he experienced several apneic episodes at home, each lasting for 30–120 s. He was rehospitalized for further evaluation. He had several paroxysmal apneic attacks in the ward, and the pulse oximetry values abruptly decreased to 22–28%. Central cyanosis and flaccidity of extremities were seen during each attack, and he recovered after tapping or lifting his torso for a maximum of 2 min. Bradycardia followed by tachycardia was detected once in two or three attacks. Although the attacks were not associated with feeding patterns, we attempted gastric decompression with nasogastric tube insertion. The frequency of episodes considerably diminished after the insertion, so we conducted further examinations. Laryngeal fiberscopy revealed no abnormality, and blood analyses, RS virus antigen test, and long-term EEG monitoring (for 5 h) were unremarkable. Upper gastrointestinal (GI) contrast study revealed esophageal motility dysfunction (Figure 1), and patient's stomach showed a gastric volvulus (GV) along its longitudinal axis (Figure 2). In a 24-h pH-probe study, the reflux index (RI) was 41.7%. With these results, GERD associated with chronic GV was diagnosed and famotidine (1 mg/kg/d) was prescribed because the parents of the boy preferred conservative treatment to surgical correction. In addition to the medication, we instructed his mother to do repeated burping with upright positioning after feeding as well as frequent small feedings. The symptoms completely subsided after therapy, and the patient was subsequently discharged.

**Figure 3:**
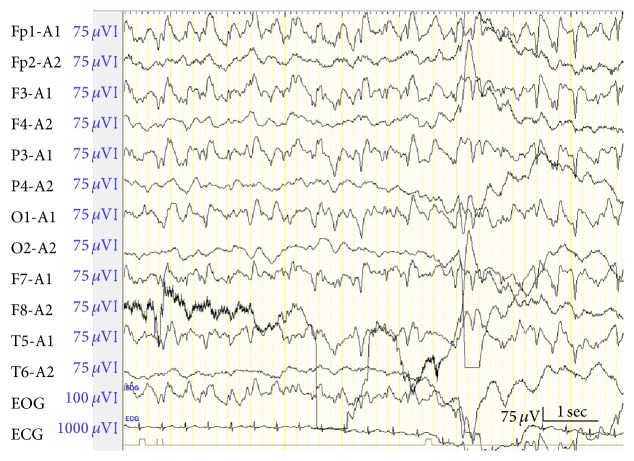
An ictal electroencephalogram during the seizure. The spike and slow wave complexes, which synchronized with apneic attacks, in left cerebral hemisphere can be seen.

For approximately 1 month, the patient had no further episodes, but the attacks recurred at age of 6 months. He was readmitted for additional evaluations. During admission, sudden-onset attacks occurred repeatedly, accompanied with unexpected immobility and apnea, and the patient showed cyanosis of face with spasticity of the extremities. Pulse oximetry was 60%–70% and the patient fell asleep after each attack. Neither bradycardia nor tachycardia was detected during these episodes. After fasting the patient, gastric decompression with nasogastric tube insertion was performed, but it was ineffective. RS virus antigen test, short-term EEG monitoring, and magnetic resonance imaging of the brain did not provide any remarkable findings. We attempted another long-term EEG monitoring (for 8 h) and witnessed an apneic attack that synchronized with a spike and slow wave complex on the EEG (Figure 3). The synchronicity between attacks and ictal waves was confirmed several times. Because the discharge occurred from the left cerebral hemisphere, we diagnosed the patient with complex partial seizures induced by localization-related epilepsy. The attacks gradually subsided with the administration of carbamazepine (10 mg/kg/d), and the patient was discharged.

A follow-up upper GI radiography at 7 months of age revealed that the patient's GV had resolved spontaneously (figure not shown). Although the RI in the follow-up pH-probe study increased from 41.7% to 53.5%, the frequency of episodes decreased to approximately once a month. Neurological development was normal at the patient's 18-month follow-up.

## 3. Discussion

ALTEs manifest as a cluster of symptoms with varying etiologies. Because approximately 50% of ALTE cases do not have any specific diagnosis [2], detection of the underlying etiology is often challenging. Hence, two or more causative diseases are rarely diagnosed in a single patient, similar to that in our case.

GERD is the most frequent etiology of ALTE, detected in 30% of cases [3]. Infantile GERD sometimes presents as extraesophageal symptoms similar to those of ALTE. Physiological gastroesophageal reflex (GER) is seen in most healthy infants in a period in which they are particularly susceptible to ALTE. However, because GERD is defined as a troublesome symptom caused by GER [4], once an infant presents with ALTE due to GER, it should be considered as GERD and should be treated as soon as possible. In the present case, during the second admission we conducted pH-probe study which resulted in extremely high value of RI [5]. We diagnosed him with GERD and initiated an empiric pharmacotherapy with H2 histamine antagonist because infants diagnosed with GERD are more likely to develop recurrent ALTE [6]. Although clinical remission was achieved, RI was not improved in the subsequent follow-up pH study. Such clinical course raises a fundamental issue regarding whether pH-probe study is an adequate modality for diagnosing GERD. pH-probe study can only evaluate acid reflux, but it cannot distinguish pathological GERD from physiological GER. Recently, this method is mentioned not to be specific enough in diagnosing GERD [7], although it was gold standard previously. Clinicians should make a strict interpretation of its results while taking into consideration the merits and limitations of this method.

In the present case, we also detected an organoaxial GV via upper GI contrast study. GV, occasionally called gastric malrotation, is a condition where all or part of the stomach rotates around either a longitudinal (organoaxial) or a vertical (mesenteroaxial) axis by at least 180° to cause total or partial obstruction of stomach on acute, intermittent, or chronic basis [8]. The symptom of GV varies depending on the extent of gastric rotation and obstruction. As for chronic GV, because it sometimes develops subtle or even asymptomatic manifestation, the diagnosis of it is so challenging for clinicians that it may be delayed for months or may remain undetected in some cases. With regard to treatment of chronic GV, currently no consensus regarding conservative or surgical management is established [9]. The GV in the present case was speculated to be intermittent or chronic on the basis of the clinical course and symptoms. We managed the chronic GV conservatively and confirmed it to have remitted spontaneously at the 7-month follow-up GI contrast study. We assumed that preventing aerophagia by lifestyle modification with frequent belching and upright positioning might contribute to symptom amelioration, because intestinal distention induced by aerophagia aggravates volvulus by pushing the greater curvature of the stomach upwards [10]. Not a few authors advocate that GV is associated with GERD [8–14]. A patient described in Al-Salem's report presented apneic spells, and GV with severe GER was detected through barium contrast study. In this case similar to ours the patient was managed conservatively, and the author stated that GER secondary to GV would disappear spontaneously once GV was corrected [10]. In another literature, Cribbs et al. stated that there may be some patients with chronic GV who are not diagnosed and treated with antireflux therapy as GERD [9]. Based on the above we assume that, in the present case, GV associated GERD was the main cause of ALTE and that clinical remission was attributed mainly to spontaneous correction of the GV irrespective of reflux acidity. We also believe that there may be potentially large infant population with undiagnosed chronic GV and that part of them may present with ALTE in which no etiology is found. Therefore, clinicians should keep chronic GV in mind as a differential diagnosis for ALTE with undetermined etiology.

Epilepsy is not a rare ALTE etiology, being the etiology in 10% of cases [2, 3]. However, it is rare to see apnea as the only clinical manifestation of epilepsy [15]. The exact mechanism of respiratory suppression in epilepsy has not been elucidated, but apnea is theoretically considered to be a result of the upper respiratory tract obstruction, respiratory muscle spasm, or respiratory effort suppression.

Tieder et al. reported that recurrent ALTEs indicate a risk for future adverse events and/or serious underlying diagnoses [16]; therefore, it is necessary for clinicians to detect pathophysiological mechanisms of recurrent ALTEs and to treat them as soon as possible for preventing hypoxic encephalopathy. In the present case, ALTE attacks recurred during the patient's third admission despite the treatment for GV associated GERD. Although EEG studies are considered to have a low sensitivity for detecting the etiology of ALTE [2], we repeated EEG studies because we suspected epilepsy from the patient's symptoms, which seemed slightly different from those perceived during his second admission. Doshi et al. reported three ALTE cases where the primary diagnosis had been GERD and additional diagnoses were made later. Of these three cases, the use of EEG contributed to the diagnosis of only one case [17]. It is time consuming and difficult to identify ictal waves on an ordinary EEG recording during a seizure, but Fu and Moon suggested that EEG be attempted in recurrent ALTE cases [2]. Hence, long-term EEG or video EEG is worth attempting when frequent events suggestive of epilepsy persist. Riquet et al. reported that the detection rate of 24-h EEG monitoring is 50% in cases where the usual frequency of the observed events is greater than once a week [18]. However, video EEG is not widely available; therefore, we recommend that clinicians who attend to intractable cases of recurrent ALTEs in which the frequency of episodes is greater than once a week refer them to specialized hospitals. Currently, there are no guidelines regarding when to initiate a prolonged or video EEG, and a prospective multicenter study for these EEG procedures is needed.

As a limitation, we cannot exclude the possibility that the patient's epilepsy had developed during the second hospitalization because the patterns of childhood epilepsy often change with time course and it is not easily detected by ordinary EEG study. In addition, the synchronicity between attacks and ictal waves in the present case was only witnessed by the attending physician. Hence, because a video EEG was not available in our ward, no objective records were available to indicate that the apneic attacks were caused by the epileptic seizures.

In conclusion, we presented ALTE case with comorbid GV associated GERD and epilepsy. Clinicians should bear in mind that there may be two or more etiologies in intractable cases of recurrent ALTEs.
